# Sir Perceval A. Nairne: Our Sailors and Their Benefactors


**Published:** 1916-07-22

**Authors:** 


### SIR PERCEVAL A. NAIRNE.

### OUR SAILORS AND THEIR BENEFACTORS.*

It would be a revelation to the world outside and to Londoners within it if we had a daily newspaper which recorded everything of importance which took place each day in the City. Far too few Britishers have ever devoted sufficient time to get even a glimpse of apprehension into the wonderful beauties, mysteries, ramifications of life, buildings, odd corners, and byways to be found within its confines. Off Moorgate Street, hidden away from the passers-by in that great artery of traffic, is situated a building of some architectural merit which constitutes the Temple of the Institute of Chartered Accountants. Its entrance and staircase lend importance to the interior, and the Council Chamber on the first floor is remarkable from the circumstance that its plan and equipment are so designed as to give the appearance of being the work of a master of hieroglyphics rather than a master of architecture. The plan might have come from the Pyramids, where many astronomical instruments have been found, and it was quite a discovery for men of artistic sense who entered the building on the 13th instant when Sir Perceval A. Nairne, chairman of the Seamen's Hospital Society, was presented with a replica of his portrait in this year's Academy.

The Accountants would appear to have degrees of precedence, if we may judge by the seating arrangements of the Council Chamber, and the opportunity afforded for providing prominent places for the lords among them. Mr. Pietro J. Michelli, the secretary of the Seamen's Hospital, acted as grand master of the ceremonies, and he was quick to note the structural opportunity provided by directing one of the vice-presidents, Sir William Bennett, K.C.V.O., to change his seat in the forum and occupy one in the Peers' Gallery, from which a few minutes afterwards he skilfully moved him into the chair. The audience filled the chamber. It was representative and full of interest from its completeness (to which Sir Perceval Nairne bore testimony in his speech), indicating the striking forcefulness of his career in attracting and winning the affections of men of all classes, professions, and occupations. Money, as a distinguished financier once remarked to the writer, is the motive power which brings us men as workers into the City of London. We do not come here to play; we come here to make money. It was well, therefore, that the Church should be represented, though it was matter for regret that the Bishop of Southwark, who desired to testify, could not attend.

We bring out these points that the hospital world may recognise that, whether by accident or design, the atmosphere must have been wholly congenial to Sir Perceval Nairne, to whom the occasion was one not only of importance but of genuine pride and heart-moving recollections, we have no doubt. For forty-two years we have had the high privilege and ever-increasing satisfaction and gain of being associated in many enterprises and continuous work of almost every kind with Sir Perceval Nairne. There are few better men to be met with in the City of London, and few indeed who have worked in the City for over half a century and won deservedly absolute confidence and very often the affection of all with whom they have been brought into intimate relations. The City, we often fancy, is as ignorant of its really great men as the citizens are of the greatness of their City. The underlying strength and supreme value of much of the life-work of Sir Perceval Nairne lies in its unostentatious, accurate, impartial thoroughness. He was described at the meeting, with truth and force, as a man to be found sitting as a member of a representative committee containing many experts, who during the greater part of the sitting would remain quiet and unnoticed during discussions which might give rise to such a difference of opinion and the evolution of so much heat as to threaten a tumult. Then suddenly a quiet little man would arise, would tersely and quickly summarise the points of the discussion, submit a short resolution dealing with everything that was material, and suggest that it might possibly meet with the acceptance of those present. This intervention would come like a ray of sunlight out of a thundercloud. Peace was established. The resolution was adopted unanimously, and everybody left for home with feelings of intense satisfaction that they had individually done much to forward the work of the nation.

The proceedings commenced by Sir William Bennett introducing Captain A. W. Clarke, deputy-chairman of the Seamen's Hospital (late *Dreadnought*), and an Elder Brother of the Trinity House. Captain Clarke's privilege was to present the replica of the portrait by Mr. George Henry, A.R.A., to Sir Perceval Nairne, a reproduction of which appears on p. 381 of this issue.

### Captain A. W. CLARKE.

It is my proud privilege and most pleasant duty to present to you, Sir Perceval, this portrait of yourself.

The original, sir, will be hung in the Board Room of the Seamen's Hospital Society at Greenwich, where it will be a reminder to the many boards of management that will follow us in the future of the good work you have done and of the esteem in which you were held by your colleagues and friends. This replica will serve, I hope, the same purpose in your own circle for those who come after you. I have served with you for some sixteen years and I never heard you say an unkind word to any man, and, better still, I have never heard you say an unkind word of any man behind his back. I have only

* The life history of Sir Perceval's and the Nairne family's connection with the *Dreadnought* will be found on pages 93 to 95 of The Hospital for April 29, 1911.

found you wrong in a few cases, and that was when I had the temerity to differ with you. It is, I believe, a cult with our modern painters to seek below the surface and try to bring to light, on canvas, the secret springs of a man's nature. They look under charity and find ostentation, under energy and find vanity, under power and find self-seeking.

Well, sir, I do not doubt that Mr. Henry, who is so great an artist, has winnowed your soul according to the latest methods, and the result is as we see it : the setting forth of our wise, dignified, and honourable chairman, the portrait of one who, in the best sense of the word, stands for the type of an English gentleman. If I may be allowed to say so, sir, it is a relief to come here to-day to honour a citizen of this country for work that is wholly constructive, and not destructive in its intentions and effects. The acclaim of the hour is, very properly, going to those who are most expert in taking life : with how much gratefulness, therefore, do we not meet here to honour the chairman of a great hospital, whose years of voluntary service have been given to bring health and longer days to his kind.

The Spirit of the Dreadnought.

The first object of our hospital is the welfare of the seamen, and your family connection with the sea, as well as your natural sympathies, have made this object specially dear to you. After all, sir, the seal of your service is not the gift we are offering you to-day : it is a far finer and wider thing. It is the common spirit that, permeating from its chairman downwards through the hospital, has concentrated itself on the purpose of healing and helping, of equipping broken men again for the service of the sea, of sending out with our discharged patients the abiding memory that the *Dreadnought* Hospital is the sailors' unfailing friend. We are transient things, sir : "We come like water, and like wind we go." Only those live on who have built themselves into the fabric of the lives of their fellow-men. No one has a fuller opportunity of doing this than the chairman of a great hospital, but he must be the true chairman, such as you are, whose counsels are wise and whose actions are prudent, who guides into the most practical and useful channels the assistance and counsels of his board. Such a chairman the *Dreadnought* possesses, and we and your friends show themselves sensible of the value of that privilege by the presentation that is being made to you to-day, and which, speaking for all your friends, I have the pleasure of offering for your acceptance. I also have pleasure in handing to you a book containing the names of all those who have joined together to do you honour, and I wish for them and for myself that you may long live to preside at our board and to enjoy the esteem, respect, and love of those who know you.

### Sir PERCEVAL NAIRNE.

Sir Perceval Nairne, in acknowledging the presentation, expressed his gratification at finding in the list of contributors which had been handed to him the names of many old and valued friends with whom he had been associated in many connections extending over a long range of years. Beginning with the Seamen's Hospital Society, and its great descendant the London School of Tropical Medicine, he recalled that he had served on the committee of management of the Society for forty-seven years, and that he had continued the services of ancestors who had been members of the committee from the time of the foundation of the Society, whilst since he joined the committee several others of the family had been appointed to it. At the commencement of his service the Society had one hospital only, with about 200 beds, in H.M.S. *Dreadnought* moored in the Thames off Greenwich and Deptford, in the great stern cabin of which he attended meetings of the committee. Now the Society's operations comprised two hospitals, two dispensaries, and two schools of medicine, in addition to which he might add a school of nursing.

Connected with his hospital work he found among the contributors representatives of the Royal National Pension Fund for Nurses and of the League of Mercy, two beneficent institutions with which he had been associated ever since their inception, and which owed their origin and their successful development to Sir Henry Burdett, to whom they also were indebted for the foundation and progress of that great achievement, the King's Hospital Fund. In both the Pension Fund and the League of Mercy Sir Henry had been told at the outset that success was impossible; that nurses were too poor and too ill-paid to insure themselves, and that small payments could never be obtained from the poorer classes, who mainly received hospital benefits, to make the League a real force. At this time the Pension Fund had an invested capital exceeding £1,800,000, while the League had paid over to the King's Hospital Fund sums amounting to £230,000.

A Many-Sided Life.

It was a matter of great gratification to him to find the Worshipful Company of Drapers among the contributors. He had held the post of solicitor to the Company for thirty-one years, and had been very proud to have had in his professional capacity a small hand during that period in the Company's great educational works, which were so well known and so highly valued in the City of London. And on the educational side also the National Union of Teachers were among his supporters. He had been connected with the Union as their general solicitor for thirty years, and from even an earlier date, and had seen their membership increase from comparatively small numbers to its present roll of between 90,000 and 100,000. With the London Teachers' Association also he had been connected for many years while its membership had been gradually increasing to a roll of more than 20,000. He found also on the list, and present in the room, many brethren of the Masonic connection in which his own career extended over more than half a century. He valued very greatly their fraternal support. It was also a pleasure to him to see representatives of his native parish of St. Giles's, Camberwell, in which he had resided all his life, and among them some old friends who were his choir-boys when he was choirmaster of the parish church from thirty-five to forty-five years ago.

He thanked the committee, the treasurer, the secretary, and all who had been concerned in the presentation for their labours in his honour and for having placed him in the hands of so genial a friend as Mr. George Henry, A.R.A., the artist, whom he also cordially thanked for his patient forbearance and consideration throughout the sittings, and for his skill in producing a portrait which was so much admired. It would be greatly valued by himself while life was continued to him, and by those of his family to whom it would descend after his time.

